# Chronic Lung Function Decline in Cotton Textile Workers: Roles of Historical and Recent Exposures to Endotoxin

**DOI:** 10.1289/ehp.0901178

**Published:** 2010-08-25

**Authors:** Jing Shi, Amar J. Mehta, Jing-qing Hang, Hongxi Zhang, Helian Dai, Li Su, Ellen A. Eisen, David C. Christiani

**Affiliations:** 1 Department of Safety Engineering, China Institute of Industrial Relations, Beijing, China; 2 Department of Environmental Health (Environmental and Occupational Medicine and Epidemiology Program), Harvard School of Public Health, Boston, Massachusetts, USA; 3 Shanghai Putuo District People’s Hospital, Shanghai, China; 4 School of Public Health, University of California Berkeley, Berkeley, California, USA; 5 Pulmonary and Critical Care Unit, Massachusetts General Hospital, and Department of Medicine, Harvard Medical School, Boston, Massachusetts, USA

**Keywords:** byssinosis, China, chronic obstructive lung disease, cotton textile workers, occupational health, organic dusts, pulmonary function

## Abstract

**Background:**

Long-term occupational exposure to cotton dust that contains endotoxin is associated with chronic respiratory symptoms and excessive decline in forced expiratory volume in 1 sec (FEV_1_), but the mechanisms of endotoxin-related chronic airflow obstruction remain unclear.

**Objective:**

In the current study, we examined temporal aspects of the exposure–response relationship between airborne endotoxin exposure, longitudinal change in FEV_1_, and respiratory symptoms in a cohort of Chinese cotton textile workers.

**Methods:**

This prospective cohort study followed 447 cotton textile workers from 1981 to 2006.at approximately 5-year intervals. We used a generalized estimating equations approach to model FEV_1_ level and respiratory symptoms as a function of past exposure (cumulative exposure up to the start of the most recent 5-year survey interval) and cumulative exposure (within the most recent interval) to endotoxins, after adjusting for other covariates. Models were stratified by active versus retired work status and by years employed before the baseline survey (< 5 and ≥ 5 years).

**Results and conclusions:**

Past exposure to endotoxin was associated with reduced FEV_1_ level among retired cotton workers. Among all cotton workers, past exposure was more strongly associated with reduced FEV_1_ for those hired < 5 years before baseline than for those who were hired ≥ 5 years after baseline. Recent endotoxin exposure was significantly associated with byssinosis, chronic bronchitis, and chronic cough.

Occupational exposure to cotton dust can cause acute respiratory responses such as chest tightness and bronchoconstriction (Merchant et al. 1973) and respiratory disease including byssinosis (Schilling et al. 1956). Longitudinal studies also indicate that long-term exposure to cotton dust may lead to chronic respiratory disease and excessive loss of pulmonary function (Beck et al. 1982; Christiani et al. 2001; Glindmeyer et al. 1991). Experimental and observational studies also suggest that bacterial endotoxin, present in cotton dust, may be a major causative agent contributing to airway inflammation and obstruction (Castellan et al. 1987; Christiani et al. 2001; Rylander et al. 1989). Generally released from bacterial lysis, endotoxins are ubiquitous in the airborne environment, but high airborne-endotoxin levels are observed in industrial environments, such as cotton mills, waste collection sites, swine barns, and grain handling equipment, where organic dusts are present (Liebers et al. 2006).

The acute respiratory response to occupational exposure to endotoxin has been described in longitudinal studies based in the cotton textile and agricultural industries (Mandryk et al. 1999; Wang et al. 2003a) However, the long-term exposure–response relationship between endotoxin and change in lung function and respiratory symptoms is not well understood. A 3-year longitudinal study of pig farmers observed a significant linear association between endotoxin exposure and annual decline in forced expiratory volume in 1 sec (FEV_1_). In fact, a doubling of exposure was associated with a 19-mL increase in the average annual decline of FEV_1_ (Vogelzang et al. 1998). Previous analyses of the present study population, a cohort of Chinese cotton textile workers, evaluated longitudinal change in FEV_1_ and the occurrence of chronic respiratory symptoms in relation to cumulative endotoxin exposure. Significant associations with longitudinal change in FEV_1_ and respiratory symptoms were identified for endotoxin exposures at 15 and 20 years of follow-up (Christiani et al. 2001; Wang et al. 2005). Whether the excessive decline in lung function and the occurrence of respiratory symptoms are influenced differently by more recent or more remote exposure to endotoxin is unknown.

The timing of exposure is not the only potential influence on the long-term effects of endotoxin on changes in lung function. Cohort eligibility requirements at baseline may also affect exposure–response relations because of variation in exposure histories before the baseline survey, a factor that has been observed in other long-term prospective studies of chronic disease (Applebaum et al. 2007; Chenard et al. 2007; Le Moual et al. 2008). For example, participants who worked many years before the baseline survey may have experienced significant declines in lung function that had already plateaued prior to the baseline evaluation, whereas participants with fewer years of employment before baseline might be more likely to experience deterioration of lung function during follow-up.

In the present study, we evaluated the exposure–response relationship between cumulative endotoxin exposure and longitudinal change in FEV_1_ and the occurrence of respiratory symptoms in an updated follow-up of a 25-year prospective cohort study of Chinese cotton textile workers. We also examined whether chronic respiratory health effects were attributable to past or more recent exposure to endotoxin and whether effects of exposure on change in FEV_1_ varied by work histories prior to the baseline survey.

## Subjects and Methods

Detailed information on subject selection, methods for testing pulmonary function, and exposure assessment has been described elsewhere (Christiani et al. 1986, 1993, 2001) and is summarized below.

### Study population

The Shanghai Textile Worker Cohort Study was established in 1981. It included 447 cotton textile workers who were exposed to airborne cotton dust and endotoxin from two cotton textile mills and 472 unexposed silk workers who worked in a neighboring silk textile mill. Participants had no symptoms of respiratory disease and had worked at least 2 years in the industry before the baseline survey. Follow-up surveys were conducted in 1986, 1992, 1996, 2001, and 2006. All participants were included in the analysis, but the rates of participation at each survey varied: 85.6% in 1986, and in 1992, 74.4% in 1996, 74.9% in 2001, and 69.1% in 2006. Overall, 260 (58.2%) cotton workers and 243 (51.5%) silk workers participated in all six surveys. The institutional review boards (IRBs) of the Harvard School of Public Health, the Putuo District People’s Hospital, and the Human Resources Administration of China approved the study. We complied with all applicable requirements of the United States and international regulations (including IRB approval); the participants gave written informed consent before the study.

### Exposure assessment

Stationary measurements of airborne cotton dust were performed with a vertical elutriator (General Metalworks Corp., Mequon, WI, USA) in the two cotton textile mills, in six work areas where yarn was prepared (Christiani et al. 1993). Sampling occurred during the first four surveys. Exposure measurements collected in the first survey were used to estimate pre-1981 levels. Between the 1986 and 1992 surveys, the mills began to blend synthetic fiber with cotton, and pure yarn production slowed. As a result, measurements taken during the 1996 survey showed that dust and endotoxin exposures were reduced by half relative to the preblend levels (Christiani et al. 2001). Sampling did not occur during the 2001 and 2006 surveys because the cotton textile and silk mills closed major operations before the 2001 survey (in 1997 and 1998, respectively).

To measure airborne endotoxin concentrations, endotoxin assays were performed on the cotton dust sample filters using the Limulus amoebocyte lysate assay, chromogenic method (Kinetic-QCL; BioWhittaker, Walkersville, MD, USA) (Olenchock et al. 1990). Endotoxin analyses were performed at the U.S. National Institute of Occupational Safety and Health (Atlanta, GA), and identical laboratory protocol and methods were used at each survey (Christiani et al. 1986). Geometric means of airborne endotoxin concentrations were computed for each work area in endotoxin units per cubic meter (EU/m^3^). Cumulative exposure to endotoxin (EU/m^3^-years) estimated for each participant was derived from work area samples and from detailed work histories. A limited number of full-shift samples were also taken in the silk mill, and measurements for endotoxin were nondetectable (below the limit of detection); thus, silk workers were considered unexposed to endotoxin and to cotton dust.

### Outcome assessment

Tests of pulmonary function were conducted according to American Thoracic Society (ATS) criteria and were supervised and recorded by trained technicians at the cotton mills (Christiani et al. 1986, 2001). Forced expiratory spirograms were performed before and after work shifts on the first day back to work after a 2-day rest period. In addition, all available retirees were tested in the cohort follow-up surveys. An 8-L water-sealed field spirometer (W. E. Collins Co., Braintree, MA, USA) calibrated twice a day with a 3-L syringe was used to record spirometric maneuvers throughout the surveys. Workers were asked to refrain from smoking for at least 1 hr before performing the test. Each worker performed up to seven trials to produce three acceptable curves. The study analyses focused on indices of FEV_1_. Acceptable FEV_1_ tracings varied by no more than 5% or 100 mL, whichever was greater, and all values were corrected for body temperature and pressure saturated with water vapor [body temperature, (ambient) pressure saturated]. The highest FEV_1_ values from technically acceptable tests were used in the analyses. At the 2006 survey, 30 cotton workers and 44 silk workers did not perform the spirometry testing because of poor health.

### Respiratory questionnaire

We used a modified ATS standardized respiratory symptom questionnaire (Ferris 1978), translated into Chinese and back-translated into English, to collect information on work, medical, and smoking history. In this study, we used the definitions of respiratory symptoms, including chronic bronchitis, chronic cough, and dyspnea, and of respiratory syndromes, including byssinosis, that were previously described by Wang et al. (2003a). We defined byssinosis (all grades) as chest tightness or shortness of breath at work that occurred on the first day or other days of the work week (see Schilling et al. 1963).

### Statistical analysis

We used generalized estimating equations (GEEs) (Zeger and Liang 1986) to account for repeated measures in generalized linear models for lung function and prevalence of respiratory symptoms at each survey using PROC GENMOD (SAS Institute Inc., Cary, NC, USA). We compared Akaike Information Criterion (AIC) statistics to evaluate the goodness of fit. An exchangeable correlation structure, with constant correlation between repeated measures of the outcome within each subject over time, was chosen based on AIC, from among biologically plausible candidates. We used an identity link function when the outcome was FEV_1_ level (at each survey over 25 years) and a logit link function when the outcome was chronic bronchitis, chronic cough, dyspnea, or byssinosis.

**Figure 1:**
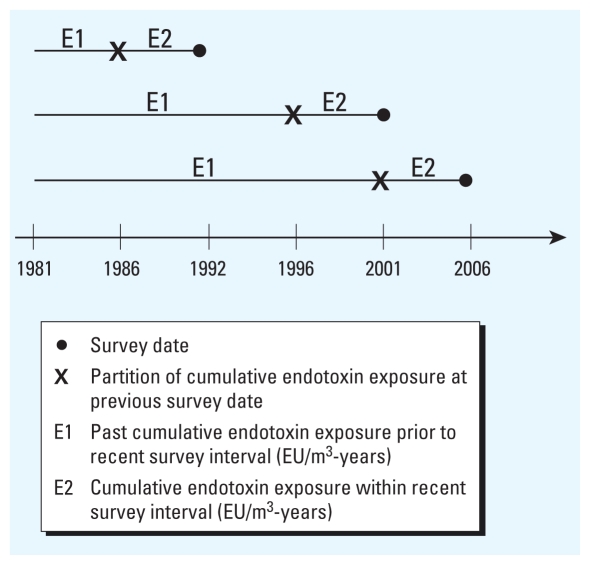
Cumulative exposure to endotoxin illustrated as time windows of exposure.

Cumulative exposure to endotoxins that was estimated at each survey was divided into two time windows: past endotoxin exposure, from date of hire up to the start of each survey interval, and recent endotoxin exposure within each current interval (Figure 1). Cumulative exposure to cotton dust was not included in the model because cumulative dust and endotoxin exposure were strongly correlated (*r* = 0.92) among the cotton workers. All models for FEV_1_ level and symptoms were adjusted for age (year), height (centimeters), sex (female as reference), smoking status (current or former smoker with never smoker as reference), work status (active vs. retired) and years since cessation of exposure. All covariates were treated as time-dependent factors except sex and height (defined as the average of the first three surveys). Years since cessation of exposure was defined as zero during active employment and as the time (years) between the date last worked in an endotoxin-exposed job in the cotton mill and the date of the current survey. Models for FEV_1_ level were stratified on employment status (active workers vs. retirees) and by years hired prior to the baseline (1981) survey (< 5 and ≥ 5 years). A *p*-value < 0.05 was selected to indicate statistical significance.

To evaluate assumptions of linearity in the exposure–response models, FEV_1_ was also modeled as a smoothed function of past endotoxin exposure, recent exposure, and years since cessation of exposure, using penalized splines in generalized additive mixed models (Eilers and Marx 1996). The degrees of freedom were selected for the smoothed terms according to the minimum AIC. R (version 2.8.1; R Foundation for Statistical Computing 2008) was used to perform the GAMM analyses.

## Results

Compared with cotton workers, nonparticipants were more likely to be men, older, and nonsmokers and to have had significantly larger annualized declines in FEV_1_ from baseline to the 2001 survey (Table 1). The average intensity of endotoxin exposure was lower at the 1996 survey than at previous surveys because the cotton mills began to blend synthetic fiber with cotton between the third (1992) and fourth (1996) surveys, and pure cotton yarn production slowed.

We modeled both recent exposure and years since cessation of exposure as continuous terms because results from penalized spline models (data not shown) suggested that associations with FEV_1_ did not significantly deviate from linearity (*p* = 0.08 and 0.45, respectively). By contrast, evidence from penalized splines suggested associations with past exposure were nonlinear. Thus we treated past exposure as a discrete variable with high-, medium-, and low-level categories that were derived from tertile cutoffs of past cumulative endotoxin exposure at each survey.

When cotton workers were examined as a whole, high and medium levels of past endotoxin exposure were associated with higher FEV_1_ relative to low level of past exposure, but only the association with the medium level was statistically significant (Table 2). A positive association was also observed between recent endotoxin exposure and FEV_1_ level among all cotton workers. FEV_1_ level increased significantly with years since cessation of exposure, which suggests that active versus retired work status might be an important confounder or effect modifier of endotoxin exposure. The association between past endotoxin exposure and FEV_1_ level was positive among active workers and negative (inverse) among retired cotton workers, whereas the association between recent endotoxin exposure and FEV_1_ level was inverse among active workers and positive among retired workers. Among all cotton workers, interactions between high and medium levels of past endotoxin exposure and retired status (*p* = 0.02, *p* = 0.52, respectively) and between recent endotoxin exposure and retired status (*p* = 0.55) were not statistically significant.

Cotton workers worked for 16.4 years, on average, before the baseline survey, and only 14.8% had worked < 5 years. We observed a stronger inverse association between FEV_1_ level and past endotoxin exposure for cotton workers who were employed < 5 years before the baseline evaluation than for workers who had worked for a longer period of time (Table 3). Significant interactions (*p* < 0.05 for high-past exposure; *p* < 0.0001 for medium-past exposure) were observed between < 5-year work tenure and high and medium levels of past endotoxin exposure.

Odds ratios for self-reported byssinosis and dyspnea were significantly increased for continuous recent endotoxin exposure (Table 4). Years since cessation was significantly associated with lower odds of byssinosis, chronic bronchitis, and chronic cough. The results were similar in a pooled model that included 472 unexposed silk workers who were also enrolled in the study (data not shown).

## Discussion

In the most recent follow-up of a 25-year prospective cohort study of cotton textile workers, we observed decrements in FEV_1_ level for retired workers with higher past cumulative exposure to endotoxin. Among all exposed workers, the inverse effect of past cumulative exposure on FEV_1_ level was largest for those with shorter work histories before the baseline survey. In contrast, recent exposure to endotoxin, rather than past exposure, was associated with byssinosis and chronic bronchitis.

When cumulative exposure to endotoxin was partitioned into two time windows of exposure, past cumulative exposure ending approximately 5 years before the survey date, rather than recent exposure to endotoxin, was inversely associated with FEV_1_ level after retirement. A lag period between exposure and disease onset has been observed for occupational exposure-related diseases such as cancer and pneumoconiosis (Checkoway et al. 1990). Lagged effects of exposure are typically detected by censoring the exposure by a fixed time period of *k* years prior to time at risk (Salvan et al. 1995). In contrast with a lagged exposure, the time windows presented in this study allowed for evaluation of past and recent exposures in the same model. The results suggest that cumulative endotoxin exposure > 5 years may influence FEV_1_ more than recent exposures over the 25-year study period, but other exposure lag periods were not assessed.

Few studies have previously investigated the different effects of historical and recent exposure to endotoxins on respiratory symptoms among cotton workers. A previous cross-sectional analysis of this population showed that a high level of current endotoxin exposure was associated with increased prevalence of chronic bronchitis and byssinosis (Kennedy et al. 1987). The results of our current study suggest that dyspnea and byssinosis may be more strongly associated with endotoxin exposure in the recent survey interval than with past cumulative exposure. Why past cumulative exposure is more strongly associated with FEV_1_ level and recent exposure is more strongly associated with respiratory symptoms of dyspnea and byssinosis is not entirely clear.

We observed a stronger association with FEV_1_ for medium than for high past cumulative exposure, and the association varied by work tenure at baseline, with the results suggesting a stronger adverse effect of past exposure among workers hired closer to the baseline evaluation. One possible explanation is that healthier workers continue to work longer than workers who are less healthy (Arrighi and Hertz-Picciotto 1994; Meijers et al. 1989). Another possible explanation for the observation is left truncation, also referred to as survivor bias, which occurs in cohort studies when an entry criterion must be met prior to start of follow-up (Szklo 1987). In occupational studies this can occur if the study population includes subjects who were hired long before the start of follow-up and who are still at work when follow-up begins. For example, Applebaum et al. (2007) found that including subjects who were hired before the start of follow-up introduced survivor bias among participants in a cohort study of Vermont granite workers. The stronger association with endotoxin among the more recently hired is consistent with such a survivor effect. That is, participants hired many years before the baseline survey may be a survivor subset of their coworkers including those no longer working. The earlier hires may also have experienced significant declines in lung function prior to baseline, with a subsequent plateau in FEV_1_ during follow-up, in contrast to those who had worked fewer years and whose deterioration in lung function was observed during follow-up. Studies of newly hired cotton textile workers have observed decrements in lung function, increased airway reactivity, and occurrence of respiratory symptoms within the first year of employment (Bakirci et al. 2007; Wang et al. 2003a, 2003b). Furthermore, workers who report symptoms tend to leave employment earlier, whereas for those who stay employed, a tolerance effect may develop (Bakirci et al. 2007).

Another explanation is that workers with longer work tenures who were available at the baseline survey may have been less susceptible to effects of endotoxin exposure compared with workers who left the industry before baseline. The findings from recent studies in cotton textile and agricultural workers suggest that the association between endotoxin exposure and reduction in lung function and occurrence of symptoms may also vary by genetic susceptibility to the inflammatory and oxidative response to the endotoxin (Hang et al. 2005; Zhang et al. 2007).

Evidence from both human exposure studies (Castellan et al. 1987; Rylander et al. 1989) and prospective epidemiologic studies (Christiani et al. 2001; Wang et al. 2005) suggest that the most likely agent in cotton dust responsible for acute and chronic pulmonary responses is gram-negative bacterial endotoxin. Previous analyses of this cohort of cotton workers also suggested that the observed associations with FEV_1_ were stronger for cumulative endotoxin exposure than for cotton dust (Christiani et al. 2001; Wang et al. 2005). Endotoxin is a potent, nonspecific stimulant of the immune system that results in adverse effects. Human and animal inhalation exposure studies of endotoxin demonstrate acute effects including airway and alveolar inflammation and decrements in lung function (Burrell 1990; Reed and Milton 2001).

However, the biological mechanism for endotoxin-related development of chronic airway disease is still unclear. An *in vitro* study of rat cells, Garcia-Verdugo et al. (2008) observed that LPS via toll-like receptor 4 induces a time-dependent increase in P2Y(2) receptors. This increase may lead to prolonged agonist-induced Ca (2+) responses that trigger higher activity in vesicle fusion and secretion. Further, these researchers suggested that long-term, but not short-term, exposure to endotoxin sensitizes alveolar type II cells, resulting in an increased extracellular surfactant pool, thus aiding pulmonary host defense mechanisms. If there is a wide range in human cellular defense responses to endotoxin, which result in acute and chronic airway inflammation, there may be variation in the time between exposure and response, as observed in our study.

The present study had several limitations. Because of the inherent limitation of the study design, surveys were conducted at approximately 5-year intervals over 25 years of follow-up. More frequent follow-up with exposure assessment and FEV_1_ measurement would have been required to evaluate lag timing of endotoxin exposure on FEV_1_ level more precisely. Selection bias may have possibly resulted from loss to follow-up, lack of participation, unacceptable pulmonary function testing, and healthy worker survivor effect. A total of 39 cotton workers and 31 silk workers died before the last survey. Participation in the questionnaire surveys was remarkably high during the 25-year follow-up. In the last survey, 30 workers participated in the survey, but they did not complete the spirometry testing, or their test results were unsatisfactory. These workers tended to be older, to have lower baseline FEV_1_, and to experience much higher cumulative endotoxin exposure. These individuals also had significantly greater FEV_1_ decline over the first 20 years of the study (57 mL/year) compared with participants who completed spirometry testing at the last survey (31 mL/year). This may represent another potential source for downward bias on the association between past cumulative endotoxin exposure and decline in FEV_1_ level.

Airborne endotoxin concentration was also estimated from sampling airborne cotton dust at fixed positions in work areas, rather than from sampling the air in the personal breathing zone of each participant. Although a reasonable correlation between personal and work area measures of airborne endotoxin has been observed in the Shanghai cotton textile industry (Mehta et al. 2008), the lack of personal air sampling data may be a possible source of exposure misclassification for this study. Moreover, air sampling of dust and bacterial endotoxin was not performed throughout the entire period of follow-up but at 5-year intervals for a duration of 3–6 months. Thus, the estimated personal cumulative exposure might not accurately reflect the actual level of individual exposure. Additionally, the LAL assay is a common assay for quantifying airborne endotoxin concentration from cotton dust samples, but there is no universally accepted standard protocol (Dungan and Leytem 2009). However, Spaan et al. (2008), in a study of sewage-derived endotoxin exposure, suggested that the LAL assay did not result in much exposure misclassification after comparison of sampling and analytical techniques. Mehta et al. (2008) also observed a high correlation in estimated airborne endotoxin concentration (log EU/m^3^) between two laboratories performing an analysis of duplicate samples of airborne cotton dust. In general, background levels of endotoxin in the environment are < 10 EU/m^3^. However, mean endotoxin concentrations in several occupational settings with airborne exposure to organic dusts or decaying organic matter have ranged from several EU/m^3^ (Bunger et al. 2007) to 7,500 EU/m^3^ (Schierl et al. 2007). The range of mean endotoxin concentrations reported in work areas of our study (Christiani et al. 1994), approximately 40–5,800 EU/m^3^, is similar to the range (~ 20–4,500 EU/m^3^) estimated in a meta-analysis of airborne endotoxin concentration in the cotton textile industry (Lane et al. 2004)

In summary, past rather than recent cumulative exposure to endotoxins before baseline FEV_1_ measurement was associated with an annual decline in FEV_1_ among retired Chinese cotton textile workers in a 25-year prospective study. Among all exposed workers, the largest decline in annual FEV_1_ associated with past cumulative exposure to endotoxin was found among those who were recently hired before the baseline survey. Additionally, recent interval exposure to endotoxin (within 5 years), rather than past cumulative exposure, was associated with chronic respiratory symptoms in retirees. The data suggest that timing of occupational endotoxin exposure may influence the rate of FEV_1_ decline as well as the incidence of respiratory symptoms among textile workers.

## Figures and Tables

**Table 1:** Demographic characteristics of participants and nonparticipants at the last survey in 2006.

Characteristic^a^	Participants	Nonparticipants
Subjects (*n*)	317	130
Men [*n* (%)]	140 (44.2)	73 (56.2)
Baseline age (year)	36.5 ± 10.0*	41.1 ± 11.4
Baseline FEV_1_	2959.6 ± 707.6	2816.2 ± 760.3
Baseline ever smoking status [*n* (%)]	121 (38.2)	37 (28.5)
Years since cessation of exposure^b^	12.7 ± 5.3**	17.7 ± 4.3
Cumulative endotoxin exposure (EU/m^3^-years)^c^	52,820 ± 45,507	61,451 ± 58,626
Intensity of endotoxin (EU/m^3^)^d^		
1981	2,086 (677–3,779)	3,003 (681–3,638)
1986	1,078 (696–5,072)	3,584 (849–5,883)
1992	1,289 (646–5,021)	1,082 (117–2,259)
1996	98 (0–878)	797 (0–1,594)
Annualized changes in FEV_1_ over the first 20 years (mL/year)^c^	−31.8 ± 16.5#	−41.3 ± 21.7

a Valus are mean ± SD, unless otherwise stated.

b Comparison made only among retired participants at the 2001 survey.

c Comparison made only among participants at the 2001 survey.

d Median (interquartile range) of work area concentration for cotton workers.

* Significant difference between participants and nonparticipants, *p* < 0.0001.

** Calculated among retired workers only, difference between participants and nonparticipants, *p* = 0.07.

# Significant difference between participants and nonparticipants, *p* < 0.01.

**Table 2:** Regression coefficients from GEE model for associations of FEV_1_ (milliliters) with endotoxin exposures among cotton workers evaluated at successive 5-year survey intervals over 25-year follow-up.^a^

	All cotton workers	Actively employed	Retired
	Estimate (95% CI)	Estimate (95% CI)	Estimate (95% CI)
Years since cessation of exposure	6.02 (0.82 to 11.21)*	NA	14.53 (6.35 to 22.71)*
Past endotoxin exposure (high)^b^	16.72 (−42.04 to 75.49)	14.12 (−41.77 to 70.02)	−132.64 (−281.11 to 15.83)
Past endotoxin exposure (medium)^b^	53.82 (10.68 to 96.96)*	39.40 (1.04 to 77.77)*	−153.56 (−312.33 to 5.21)
Recent endotoxin exposure (1,000 EU/m^3^-years)^c^	0.77 (−0.50 to 2.04)	−0.07 (−1.51 to 1.37)	3.32 (−0.91 to 7.54)

NA, not applicable.

a Estimated from GEE models using all available data from 447 cotton workers. All variables except sex and height are treated as time-dependent variables. Models were adjusted for age, mean height of the first three surveys, sex, and smoking status.

b Past endotoxin exposure was defined as cumulative exposure to endotoxin (EU/m^3^-years) before the start of the survey interval in which FEV_1_ was measured but was modeled with two binary variables comparing high with low and medium with low levels. Tertile percent cutoffs of past endotoxin exposure at each survey was used to defined low, medium, and high levels. For workers hired < 5 years before the baseline survey, past endotoxin exposure before 1981 was zero.

c Recent endotoxin exposure was defined as cumulative exposure within the 5 years prior to each FEV_1_ measurement and was modeled continuously; estimates indicate the change in FEV_1_ level associated with a 1,000 EU/m^3^-years increase in endotoxin exposure.

* *p* < 0.05.

**Table 3:** Adjusted estimates for FEV_1_ level (milliliters) over 25-year follow-up, stratified by work tenure at baseline survey.^a^

	Hired < 5 years before baseline (*n* = 66)	Hired ≥ 5 years before baseline (*n* = 381)	Hired ≥ 10 years before baseline (*n* = 285)
	Estimate (95% CI)	Estimate (95% CI)	Estimate (95% CI)
Years since cessation of exposure	−10.69 (−34.50 to 13.12)	7.30 (2.30 to 12.30)*	11.03 (5.31 to 16.76)*
Past endotoxin exposure (high)^b^	−93.13 (−204.99 to 18.72)	−30.30 (−103.63 to 43.03)	−39.19 (−128.69 to 50.32)*
Past endotoxin exposure (medium)^b^	−37.93 (−109.33 to 33.47)	27.54 (−23.15 to 78.23)	5.09 (−71.53 to 81.70)
Recent endotoxin exposure (1,000 EU/m^3^-years)^c^	−0.09 (−2.89 to 3.06)	1.16 (−0.32 to 2.63)	1.56 (−0.27 to 3.39)

a Estimated from GEE models using all available data from 447 cotton workers. All variables except sex and height were treated as time-dependent variables. Models were adjusted for age, mean height of the first three surveys, sex, and smoking status.

b Past endotoxin exposure was defined as cumulative exposure to endotoxin (EU/m^3^-years) before the start of the survey interval in which FEV_1_ was measured, but was modeled with two binary variables comparing high with low and medium with low levels. Tertile percent cutoffs of past endotoxin exposure at each survey was used to defined low, medium, and high levels. For workers hired < 5 years before the baseline survey, past endotoxin exposure before 1981 was zero.

c Recent endotoxin exposure was defined as cumulative exposure within the 5 years prior to each FEV_1_ measurement and was modeled continuously; estimates indicate the change in FEV_1_ level associated with a 1,000 EU/m^3^-years increase in endotoxin exposure.

* *p* < 0.05.

**Table 4:** Adjusted odds ratios (95% CIs) for self-reported respiratory symptoms among cotton workers over 25 years.^a^

	Byssinosis	Chronic bronchitis	Dyspnea	Chronic cough
No. of workers who ever had symptoms	139/447	132/447	194/447	122/447
Years since cessation of exposure	0.89 (0.84–0.94)*	0.95 (0.90–1.00)	0.98 (0.95–1.02)	0.93 (0.89–0.99)*
Past endotoxin exposure (high)^b^	0.87 (0.55–1.38)	1.21 (0.81–1.80)	1.07 (0.79–1.46)	1.42 (0.94–2.14)
Recent endotoxin exposure (1,000 EU/m^3^-years)^c^	1.02 (1.00–1.04)*	1.00 (0.99–1.02)	1.01 (1.00–1.03)*	1.00 (0.98–1.01)

a GEE analysis was performed using all available data from 447 cotton workers over 25 years of follow-up. All variables except height and sex were treated as time-dependent variables. Models were adjusted for age, mean height of the first three surveys, sex, and smoking status.

b Past endotoxin exposure was defined as cumulative exposure to endotoxin (EU/m^3^-years) prior to start of the survey interval but was modeled as a binary variable comparing high with low levels. The selected cut point for high level of exposure was the 75th percentile of the distribution of past endotoxin exposure at each survey. For workers hired < 5 years before the baseline survey, past endotoxin exposure before 1981 was zero.

c Recent endotoxin exposure was defined as cumulative exposure within the 5 years prior to each FEV_1_ measurement and was modeled continuously; estimates indicate the change in FEV_1_ level associated with a 1,000 EU/m^3^-years increase in endotoxin exposure.

* *p* < 0.05.
